# Midkine release during hemodialysis is predictive of hypervolemia and associates with excess (cardiovascular) mortality in patients with end-stage renal disease: a prospective study

**DOI:** 10.1007/s11255-022-03141-4

**Published:** 2022-02-24

**Authors:** Sabine Brandt, Anja Fischer, Carla Kreutze, Dorothea Hempel, Xenia Gorny, Florian G. Scurt, Delia L. Şalaru, Peter Bartsch, Anja Bernhardt, Stefanie M. Bode-Böger, Matthias Girndt, Roman Fiedler, Berend Isermann, Jonathan A. Lindquist, Peter R. Mertens

**Affiliations:** 1 Clinic of Nephrology and Hypertension, Diabetes and Endocrinology, Otto-Von-Guericke-University Magdeburg, Leipziger Strasse 44, 39120 Magdeburg, Germany; 2 Present Address: University of Medicine and Pharmacy, Gr.T.Popa Iasi, Iasi, Romania; 3 Institute of Clinical Pharmacology, Otto-Von-Guericke-University Magdeburg, Magdeburg, Germany; 4 Department of Internal Medicine II, Martin-Luther-University Halle, Halle, Germany; 5 Institute of Clinical Chemistry and Pathobiochemistry, Otto-Von-Guericke-University Magdeburg, Magdeburg, Germany; 6 Institute of Laboratory Medicine, Clinical Chemistry and Molecular Diagnostics, Leipzig University Hospital, Leipzig, Germany

**Keywords:** Midkine, Hemodialysis, Hypervolemia, Diabetes, Cardiovascular disease: biomarker

## Abstract

**Background:**

In end-stage renal disease, a high cardiovascular risk profile and endothelial damage prevails. The heparin-binding growth factor midkine stimulates neo-angiogenesis in ischemic diseases, coordinates neutrophil influx, and raises blood pressure through stimulated angiotensin synthesis.

**Methods:**

We determined changes of midkine serum levels during hemodialysis sessions under the assumption that endothelial cell-derived midkine is released. Periprocedural differences (∆midkine) were calculated and correlated with cardiovacular biomarkers and fluid status (clinical assessment, V. cava collapse, comet tail phenomenon), cardiovascular morbidities, mortality rates. Blood was collected before and after dialysis from hemodialysis patients (*n* = 171; diabetes: *n* = 70; hypervolemia: *n* = 83; both: *n* = 32).

**Results:**

Baseline midkine levels were ~ fourfold elevated compared to healthy controls (*n* = 100). Further, on average a tenfold rise was detected during dialysis, the extent of which was partially related to non-fractionated heparin application (*r*^2^ = 0.17). Inter-individual differences were highly reproducible. Hypervolemic patients responded with a less than average rise in midkine levels during dialysis (*p* < 0.02), this difference became more obvious with co-existing diabetes (*p* < 0.001 for long dialysis-free interval) and was confirmed in an independently enrolled dialysis cohort (*n* = 88). In Kaplan Meier survival curves, low delta midkine levels correlated with cardiovascular/overall mortality rates, similar to elevated uPAR levels, whereas other markers (NTproANP, galectin, tenascin-C) were less predictive. Following intervention with successful fluid removal in hypervolemic dialysis patients to optimize fluid homeostasis, midkine values increased (*p* < 0.002), which was not observed in patients that failed to decrease weight.

**Conclusion:**

Thus, for dialysis patients inadequate periprocedural midkine upregulation is linked with hypervolemia and associates with cardiovascular events.

**Supplementary Information:**

The online version contains supplementary material available at 10.1007/s11255-022-03141-4.

## Introduction

Midkine received its designation as a heparin binding factor, which was initially described as an embryonic growth factor with strong expression during mid-gestation and within kidneys. Pleiotropic functions have been identified beyond pregnancy, involving cellular survival programs, chemotaxis of neutrophils [1], and propagation of neoangiogenesis [2–4]. The potency of this specific growth factor is illustrated in experimental animal models with genetic ablation of the *midkine *(MDK) gene. Angiotensin converting enzyme (ACE) expression in human lung microvascular endothelial cells is dependent on midkine exposure [5]. Thus, midkine is a mediator of an intimate crosstalk between kidneys and lungs [6]. Midkine expression is regulated by oxidative stress and antioxidants may reduce midkine serum levels as well as angiotensin II synthesis [5]. Studies using ischemic vascular injury models suggest that midkine induces neointima formation, likely via macrophage and neutrophil recruitment, and regulates neoangiogenesis [7–9]. In this regard midkine may function as a key effector molecule linking RAAS functions, including blood pressure regulation, with fluid distribution and ischemic tissue damage.

Dialysis patients are prone to cardiovascular diseases and suffer from diverse hormonal counter- and dysregulations [10, 11]. We hypothesized that midkine regulation is disturbed in patients undergoing hemodialysis with stressed endothelial cells, and that micro- and macroangiopathy mirror endothelial damage with activated angiogenic programs and a systemic pro-inflammatory milieu. Furthermore, increased serum midkine levels following application of unfractionated heparin have been reported [12] in both dialysis patients and healthy controls; with unknown clinical significance.

Given the dichotomous cyto-protective and -injurious functions of midkine our primary aim was to analyze changes of midkine serum levels that occur during the hemodialysis procedure and test for associations to the fluid status. A variation in volume status was anticipated as measurements were performed following short (2 day) and long (3 day) dialysis-free intervals. Secondary, we performed correlative analyses by determining cardiovascular biomarkers (NT-proANP, uPAR, ADMA) and clinical parameters of fluid status (comet tail phenomenon of lungs, V. cava collapse, clinical assessment), co-morbidities, follow-up mortality over 36 months.

## Materials and methods

### Study design

The study was approved by local ethics committees (EK 73/90 MD and EK 2016–12 Hal). Two independent cohorts of dialysis patients were enrolled following providing written informed consent (*n* = 83, KfH Magdeburg, Germany; *n* = 88, KfH Halle, Germany). The exclusion criteria for the Study were as follows: presence of clinically apparent infections; active malignant diseases; myocardial infarction ≤ 12 weeks ago; poorly adjusted hypertension; lack of written consent after clarification. Exclusion criteria are based on pathophysiological involvement of midkine in the mentioned comorbidities [13]. Patients received replacement therapy thrice weekly. Clinical data were retrieved from the medical records. Fluid status was assessed by the caring physician and judged as “overloaded” when the optimal weight was exceeded by > 0.5 kg after dialysis. Ultrasound of the abdomen and quantification of the Vena cava width was performed, with > 20 mm being classified as fluid overload. A thoracic ultrasound was performed to detect comet tail phenomena, i.e., hyperechogenicity reflecting pulmonary edema. A carotid intima-media thickness > 0.9 mm was defined as increased. Cancer was defined as being current when diagnosis was < 2 years and/or chemotherapy was ongoing. Diabetes mellitus was diagnosed according to German Diabetes Society guidelines [14]. Reference values for C-reactive protein (CRP) were below 0.5 mg/dl. Patients from a second cohort (*n* = 88; mean age 65.4 years, range 26–91 years, 55 males, 33 females) were included in the study following a 3 day dialysis-free interval and clinically scored as eu- or hypervolemic.

An intervention study was designed and approved by the local ethical committee that addressed the issue of hypervolemia and regulation of midkine values with additional fluid removal. Patients with hypervolemia were advised to further reduce their body weight and approve additional fluid removal during dialysis sessions. In the patients, blood sampling was performed after a long dialysis-free interval and following the dialysis procedure on weekly intervals over 3 weeks. Midkine values were determined and the highest periprocedural midkine regulation calculated.

### Sample collection, storage and measurements

Blood was collected before and after hemodialysis (all patients were treated with high flux membranes, serum prepared immediately by centrifugation, and samples stored at – 80 °C. Healthy donor serum (*n* = 100) was collected for comparison. Serum midkine levels were quantified using a human midkine ELISA (PeproTech, Hamburg, Germany) and measured on a TECAN Infinite 200 spectrophotometer (detection range: 0.1–2000 ng/ml). Blood counts were analyzed using a Coulter Counter (Beckman Coulter).

Serum uPAR, and NTproANP levels were quantified simultaneously in a multiplex format using the high sensitivity cytokine/chemokine kit (Magnetic Luminex Screening Assay, human premixed multi analyte kit, R&D Systems) according to the manufacturer’s instructions. The detection limit for NTproANP is set at 66.2 pg/ml, for uPAR 43.2 pg/ml. Standard curves were established (271–68,820 pg/ml for NTproANP, 424–103,000 pg/ml for uPAR). Tenascin-C and galectin-3 serum levels were determined using enzyme-linked immunosorbent assays (Biozol and Affymetrix eBioscience, respectively) at 450 nm according to the manufacturers’ instructions with detection limits for tenascin-C set at 0.188 ng/ml and for galectin set at 0.29 ng/ml. Inter- and intra-assay variability is less than 10% in both assays.

Serum concentrations of (a)symmetrical dimethylarginine (ADMA, SDMA), and L-arginine were quantified as described [15].

Pre-analytic and analytic quality assurance was performed for midkine protein stability. The effects of (repeated) freeze–thaw cycles were tested. Serum samples from three dialysis patients were processed immediately and then stored at 4 °C, − 20 °C, or – 80 °C. Samples stored at 4 °C for 4 days yielded up to 34% lower midkine levels (Suppl. Fig 1A), whereas values for samples stored at either – 20 °C or – 80 °C did not differ. Two samples stored for 2 and 3 years at – 80 °C did not reveal significant changes of midkine values (Suppl. Fig 1B). Thus, long-term storage of serum at – 80 °C allows an accurate detection of serum midkine. Another concern is freeze–thaw cycles. Even a single cycle resulted in markedly reduced midkine values (Suppl. Fig 1C), thus repeated freeze–thaw cycles were precluded.

### Statistical analysis

Analysis was performed using the SPSS, GraphPad Prism, and MedCalc software. Data are presented as proportions for categorical variables and means ± SD or medians (interquartile range, 25–75th percentile) for continuous variables. For comparison of linear variables *t* tests or Mann–Whitney *U* tests were applied. Correlative analyses were conducted using Pearson (r) or Spearman (*ρ*) tests. Whenever possible the data were transformed logarithmically to achieve normal distributions. A *p* value < 0.05 was considered significant. Spearman correlation coefficients were calculated to characterize associations between biomarkers and clinical parameters. Coefficients of less than 0.4 were considered as indicating low, of 0.4–0.7 medium, and greater than 0.7 strong correlation.

For estimating the optimal cut-off points for uPAR, ∆midkine, galectin, tenascin-C, and NTproANP to predict survival or normovolemia/ hypervolemia, receiver operating characteristic (ROC) curves were calculated to quantify AUC values. Binary logistic regression analysis was used to identify associations.

Three separate multivariable Cox proportional hazards models were created to assess survival. Nonlinear continuous variables were made categorical. Wald statistics were used to assess the significance of exposure variables. The models were assessed using the Harrell C statistic and Akaike Information Criterion (AIC).

### Cluster analysis

Genesis Software 1.7.7 [16] was applied to perform heat map and cluster analysis using a *Z*-score transformation followed by hierarchical clustering with average linkage as agglomeration rule [17].

## Results

### Study population

The baseline demographics and clinical characteristics of the study participants are listed in Table 1. The mean age was 64 (± 16) years, 65% were males. Participants underwent regular dialysis for 4.1 years on average [range: 0.2–22 years]. Anticoagulants applied for hemodialysis were non-fractionated (70 patients) and fractionated heparin (12 patients), or citrate (1 patient). Co-morbidities retrieved from medical records were arterial hypertension (98%), diabetes mellitus (40%), coronary artery disease (48%), and cerebrovascular disease (21%). 45% (37/83) received ACE inhibitors or angiotensin type 1 receptor blockers (ARBs).

**Table 1:** Bibliographic data of hemodialysis cohort 1 (A) and selected laboratory values of the hemodialysis cohort 1 (B)

(A)	
Mean age (years)	64 ± 16
On dialysis since (years)	4.1 ± 3.9
Sex	
Male	54/83 (65%)
Female	29/83 (35%)
Co-morbidities	
Arterial hypertension	82/83 (99%)
Coronary artery disease	40/83 (48%)
Diabetes mellitus	33/83 (40%)
Carcinoma	4/83 (5%)
Cerebrovascular disease	17/83 (21%)
Anticoagulation	
Non-fractionated heparin: 70/82 (84%)	
2-day interval (IU)	6.222 ± 2.210
3-day interval (IU)	6.251 ± 2.180
Fractionated heparin (IU): 12/82 (15%)	
2/3 days interval (IU)	2.958 ± 2.875
Weight difference before/after dialysis	
2-day interval (kg)	2.1 ± 1.1
3-day interval (kg)	2.4 ± 1.1
Hypervolemia	51/83 (61%)
Intima media thickness [mm]	0.9 ± 0.2

(B)		
Parameter	Reference range	Mean value (range)
CrP (mg/dl)	< 5.0	1.4 ± 6.5 (0–59)
Leukocytes (Gpt/l)	Female: 3.9–10.4 Male: 3.7–9.8	6.4 ± 2.1 (2.4–14.5)
Calcium (mmol/l)	2.15–2.55	2.2 ± 0.2 (1.8–2.5)
Phosphate (mmol/l)	0.81–1.45	1.6 ± 0.5 (0.9–2.9)

### Midkine serum levels are elevated in dialysis patients and further rise during dialysis

**Fig. 1:**
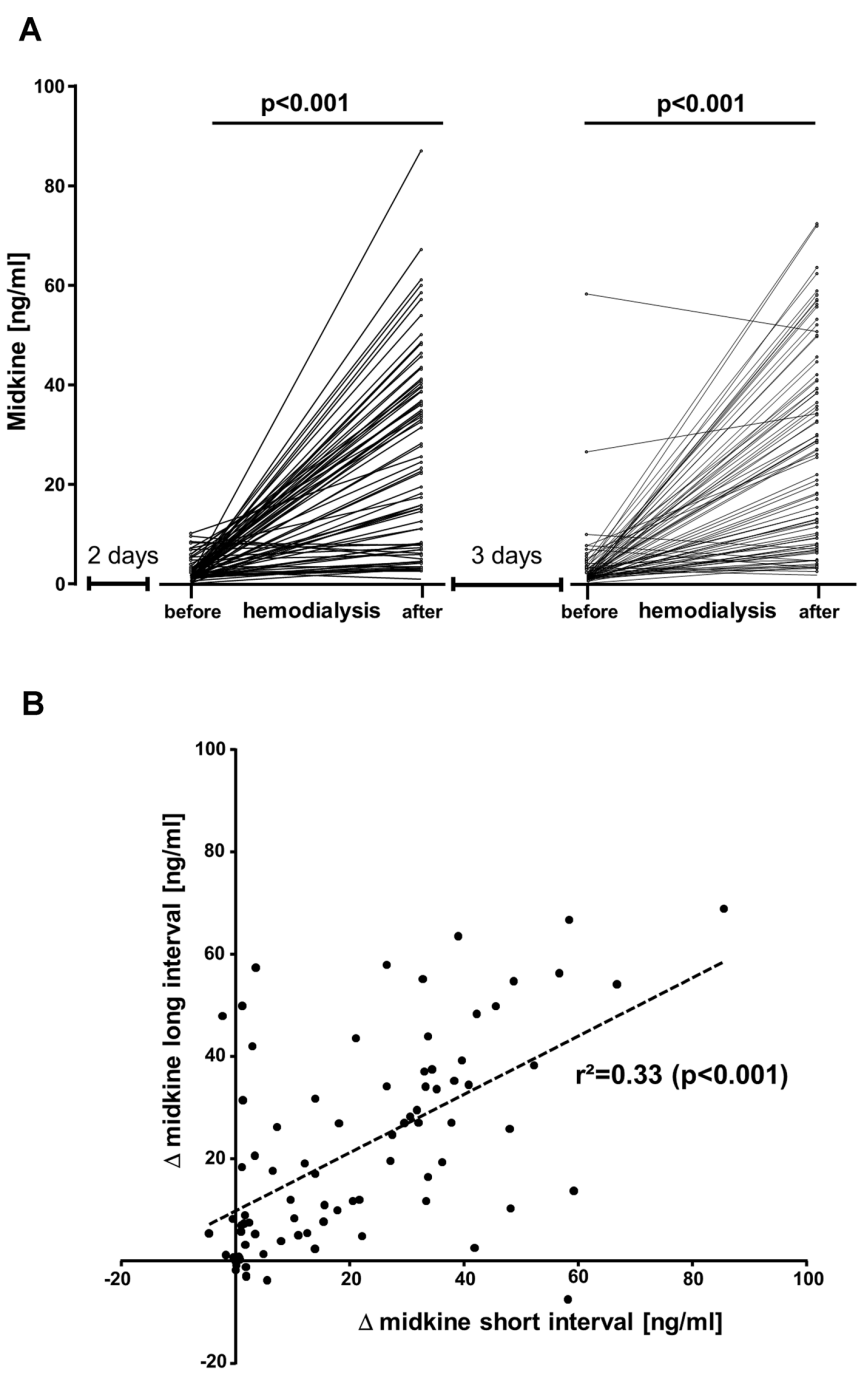
Serum midkine levels before and after dialysis treatment. All dialysis patients provided serum samples before and after two dialysis sessions following a short (2 day) and long (3 day) dialysis-free interval. **A** Individual changes in midkine serum levels are provided. Midkine serum levels after dialysis treatment increased following the short 2-day (*p* < 0.001) and long 3-day dialysis-free interval (*p* < 0.001). **B** Correlation of midkine serum level changes during dialysis after the short and long dialysis-free interval (*r*^2^ = 0.33, *p* < 0.001)

Midkine levels from 100 healthy donors were determined with a mean of 0.6 ng/ml (range < 0.1–3.6 ng/ml). For the 83 patients in cohort 1 following a short (2 day) dialysis-free interval, the mean midkine levels were 2.3 ± 2.3 ng/ml (range < 0.1–10.1 ng/ml) before dialysis and 23.4 ± 19.8 ng/ml [range 0.6–87.1 ng/ml] after dialysis. Following a long (3 day) dialysis-free interval, the mean midkine levels were 3.1 ± 6.9 ng/ml (range < 0.1–58.2 ng/ml) before and 25 ± 19.8 ng/ml (range 0.5–72.3 ng/ml) after dialysis (Fig. 1A). Thus, midkine concentrations rise significantly during the hemodialysis procedure (Fig. 1A), however not uniformly, given that some patients exhibited lower midkine levels post dialysis. Predialysis absolute midkine values did not significantly differ for 2- and 3-day intervals. To visualize the peri-procedural changes, delta (Δ) midkine values (i.e., midkine after dialysis–before dialysis) were calculated and correlated for both short and long dialysis-free intervals, yielding a correlation coefficient of *r*^2^ = 0.33 (*p* < 0.001, Fig. 1B).

### Midkine serum levels are upregulated by non-fractionated, but not fractionated heparin

Midkine is reported to be released by endothelial cells in dialysis patients and healthy volunteers following non-fractionated heparin application [12]. In our study, serum midkine levels were compared in patients receiving non-fractionated heparin (*n* = 70) with those receiving fractionated heparin (*n* = 12). Unfractionated heparin was applied following the short (6222 ± 2210 IU) and long (6251 ± 2180 IU) dialysis-free intervals. The increase in serum midkine (expressed as Δmidkine values) during dialysis exhibited an association with the administered non-fractionated heparin doses following both the short (*r*^2^ = 0.06, *p* = 0.03) and long (*r*^2^ = 0.17, *p* < 0.001) intervals, however the increase was not uniformly seen and not present with fractionated heparin (Suppl. Fig 2A, B).

### Indicators of systemic inflammation (CRP, leukocytosis), a. carotis intima-media thickness, RAAS inhibitor intake and serum midkine levels

Correlation analyses were performed to assess whether serum midkine levels are associated with indicators of systemic inflammation (Table 1B). The assessment of Δmidkine levels in relationship to C-reactive protein (CRP) (short interval *p* = 0.44; long interval *p* = 0.19) and leukocytosis (short interval *p* = 0.17; long interval *p* = 0.07) showed no correlation. Intima-media thickness also did not correlate with absolute midkine levels (data not shown). Studies with mice indicate that midkine synthesis and secretion is regulated by RAAS [5, 18, 19]. Given that 45% of the patients received RAAS inhibitors we analyzed intergroup differences. The comparison revealed that RAAS blockade did not alter midkine serum levels in dialysis patients (data not shown).

### Serum midkine levels in diabetic versus non-diabetic patients

**Fig. 2:**
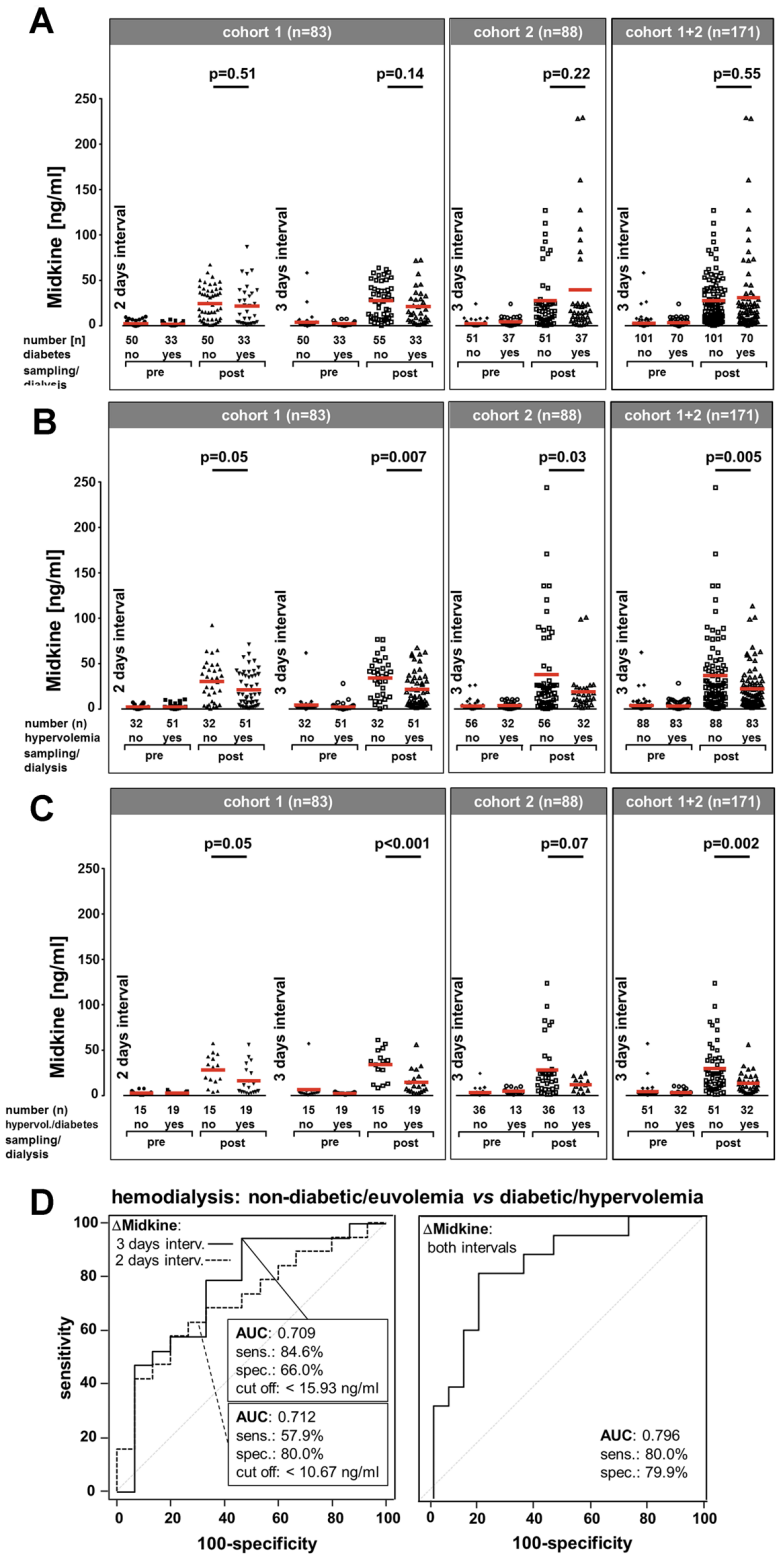
Changes in serum midkine levels during dialysis in patients diagnosed with diabetes and/or hypervolemia. **A** For the diabetic (*n* = 33) versus non-diabetic (*n* = 50) patients in cohort 1 subgroup analyses were performed. Absolute midkine values were assessed after a short and long dialysis-free interval. Intergroup comparisons did not yield significant differences. A similar analysis was performed with cohort 2, collected independently, yielding no significant difference. **B** For the patients diagnosed with hypervolemia (*n* = 51) versus euvolemia (*n* = 32) subgroup analyses were performed. Absolute midkine values were assessed after a short and long dialysis-free interval. Intergroup comparisons yielded differences after the short (*p* = 0.05) and long interval (*p* = 0.007) in cohort 1, for cohort 2 the difference was also significant (*p* < 0.05). **C** For the patients diagnosed with diabetes and hypervolemia (*n* = 19) *versus* those without diabetes and euvolemia (*n* = 15) subgroup analyses were performed in both cohorts. Absolute midkine values were assessed after a short and long dialysis-free interval. Intergroup comparisons yielded significant differences after the short (*p* = 0.05) and long interval (*p* = 0.001) in cohort 1, whereas a clear trend was seen in cohort 2 (*p* = 0.07). (**D**) Receiver operating characteristic (ROC) analyses allowed us to discriminate between non-diabetic/euvolemic versus diabetic/hypervolemic patients by means of Δmidkine values

Within cohort 1, 40% (33/83) patients were diagnosed with diabetes mellitus [14]. A statistical analysis for absolute and Δmidkine intergroup differences in diabetics *versus* non-diabetics revealed no significant differences, which was confirmed in a second cohort (*n* = 88; Fig. 2A). To assess the variability due to dialysis, ΔΔmidkine values were calculated, for the whole cohort and the two subgroups separately. Values are depicted for diabetics *versus* non-diabetics (Suppl. Fig 3A, B).

### Serum midkine levels and fluid volume status: clinical signs of hypervolemia inversely correlate with midkine values

Hypervolemia was assumed when (1) the actual body weight exceeded the clinically defined optimum by > 0.5 kg after dialysis, (2) the Vena cava diameter was > 20 mm wide or (3) a lung comet tail phenomenon was demonstrated (Suppl. Table 1). Accordingly, 61% (51/83) dialysis patients were classified as hypervolemic and the remainders as normovolemic. Given that reports suggest that vascular changes result in midkine release from endothelial cells within the lung or kidneys we correlated fluid alterations observed inter- and intra-individually with serum midkine changes. Our study protocol included fluid removal of 2.1 ± 1.1 (2 days) and 2.4 ± 1.1 L (3 days dialysis-free interval). Post-dialysis midkine values in hypervolemic patients were 20.1 ± 18 ng/ml and 20.4 ± 18.3 ng/ml, respectively. In normovolemic patients, midkine values were 28.7 ± 21.5 ng/ml and 32.3 ± 20.1 ng/ml, respectively. Hence, the mean midkine values in hypervolemic patients are significantly lower after dialysis than in patients classified as normovolemic (Fig. 2B, Suppl. Fig 3C). Similar results were determined with cohort 2.

### Diabetic patients with hypervolemia versus non-diabetics with normovolemia: correlation with dialysis-related midkine response

**Fig. 3:**
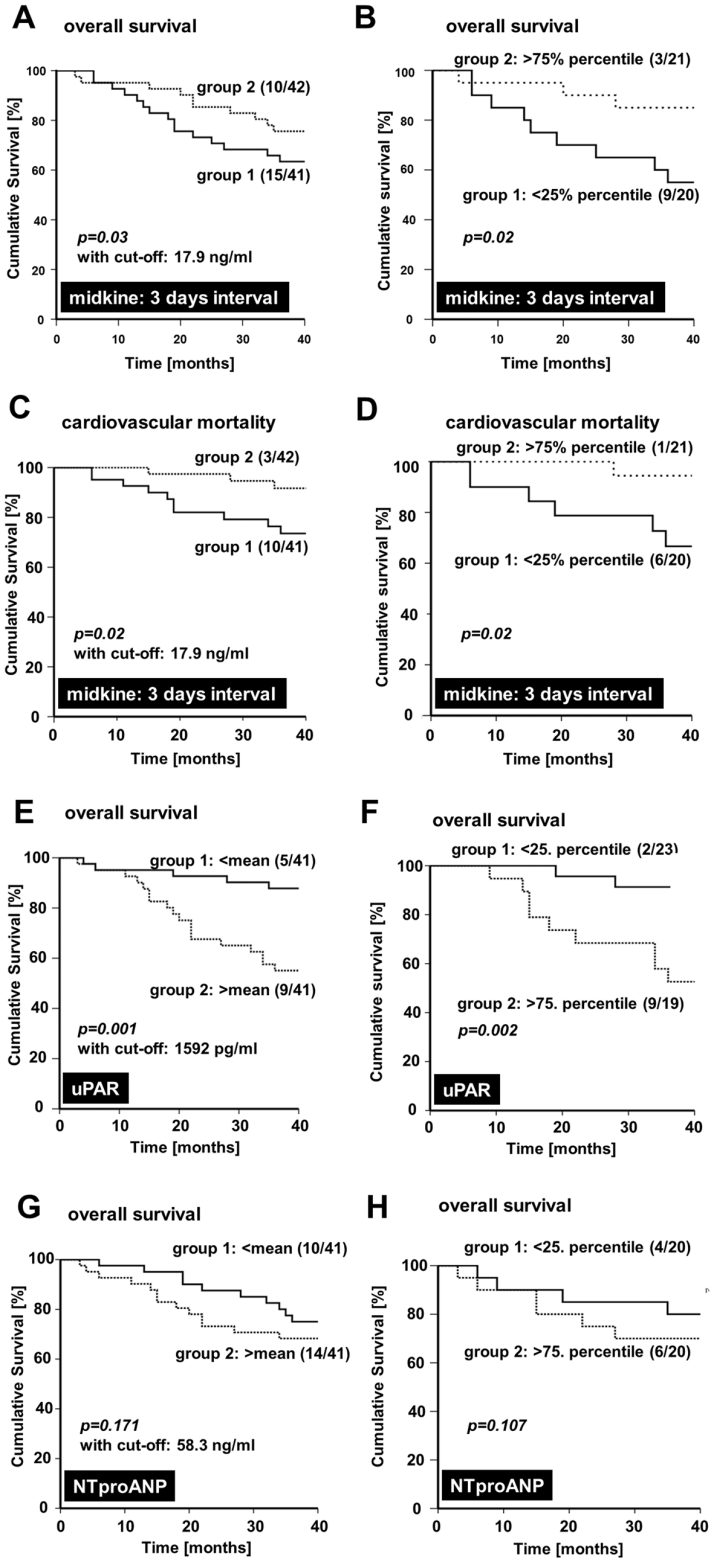
Predictive value of serum markers for overall and cardiovascular survival. Serum determinations of midkine (**A-D**), uPAR (**E**, **F**) and NTproANP (**G**, **H**) were performed and levels below average after the 3 days dialysis-free interval (group 1) were compared with those above average (group 2), similarly calculations with 25 and 75 percentile values were done. Censoring for overall as well as cardiovascular survival over a 36-month period is depicted by Kaplan–Meier curves

23% (19/83) of patients diagnosed with diabetes mellitus were hypervolemic. These constitute a high risk group for cardiovascular disease and high morbidity. For this subgroup, mean serum midkine levels after dialysis were 15.8 ± 17.1 ng/ml (2 days) and 14.0 ± 14.9 ng/ml (3 days interval). 22% (18/83) of patients were neither diabetic nor hypervolemic. For this subgroup with a lower cardiovascular risk profile, the mean midkine values were 28.2 ± 18.1 ng/ml (2 days) and 33.7 ± 18.6 ng/ml (3 days; Fig. 3C) post dialysis. Comparing midkine values for the aforementioned subgroups provided significantly lower post-procedural midkine levels in hypervolemic diabetics (Fig. 2C).

Given these compelling findings we extended the collection of serum samples to an independent cohort from other dialysis centers. Here, serum samples were collected before and after dialysis only after a 3 day long dialysis-free interval, clinical fluid homeostasis assessment was performed as in the previous cohort. The age and sex distribution in this cohort was similar to the first one. The midkine serum determinations were performed independently and the results for cohort 2 are provided in Fig. 2A, C. Again, fluid excess was accompanied by a lower midkine level post dialysis (*p* = 0.03). The results confirm a highly significant association of fluid imbalance with lower than average midkine elevation in both cohorts (*p *< 0.002), which was especially prevalent in diabetics. The calculated AUC by receiver operator characteristics for hypervolemia in diabetics versus euvolemia in non-diabetics was 0.709 after a 3 day dialysis-free interval and 0.796 (sensitivity 80%, specificity 79.9%) for both intervals combined (Fig. 2D; AUC for other combinations are provided in Suppl. Fig. 3D and E).

### Prediction of adverse cardiovascular outcome by serum Δmidkine and markers of endothelial dysfunction

Serum sample collection began in 06/2012 and patients were followed-up to 48 months. By 06/2015, 30% (25/83) of hemodialysis patients from cohort 1 had died, 13 (16%) due to cardiovascular events (sudden cardiac death, myocardial infarction, stroke). At the time of recruitment, the deceased patients had significantly lower midkine values after hemodialysis (2 days interval: 20.7 ± 17.9 ng/ml; 3 days interval 18.6 ± 15.8 ng/ml) compared to the living patients (2 days interval: 24.6 ± 20.5 ng/ml; 3 day interval: 27.7 ± 20.8 ng/ml). A classification of midkine values above/ below the mean for all dialysis patients (*n* = 83) and subgrouping revealed that low midkine levels after hemodialysis constitutes a significant adverse prognosis marker for overall (Fig. 3A, B) and cardiovascular mortality (Fig. 3C, D). For the latter group, the mean post-dialysis midkine values were 16.8 ± 15.7 ng/ml (2 days) and 13.4 ± 13.6 ng/ml (3-day dialysis-free interval).

To compare the utility of midkine serum level changes to predict cardiovascular death and overall survival additional biomarkers were selected and determined in pre-dialysis serum samples. These were uPAR [20], NTproANP, galectin [21] and tenascin-C [22], all of which have been linked to adverse clinical outcome in hemodialysis patients. The obtained results confirm the predictive value of uPAR, however refute the utility of the remainder (Fig. 3E, H; Table 2; Suppl. Fig. 4A–D). Notably, NTproANP as a marker for cardiac stress was of inferior predictive value for overall and cardiovascular survival in the dialysis cohort. Analyses revealed that pre-dialysis NTproANP and absolute midkine values pre-dialysis in serum samples correlated in diabetic dialysis patients (*r* = 0.6), whereas such a correlative link is much weaker between midkine and uPAR serum levels (Suppl. Fig 5A–D).

**Table 2:**
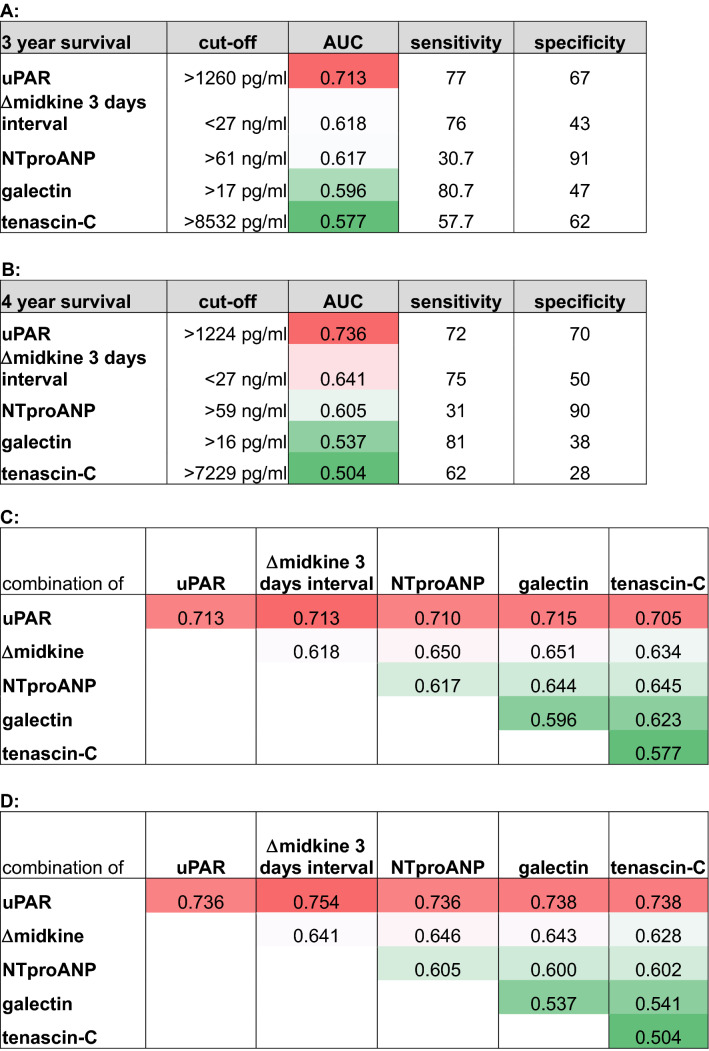
Receiver operating characteristics of serum markers to predict cardiovascular survival

Selected serum markers were tested for prognostic sensitivity and specificity to foresee survival at 36 and 48 months. (A, B) Area under the curve (AUC) values obtained by serum markers to predict 3- and 4-year survival. Cut-off values were selected to yield the indicated sensitivity and specificity values. (C, D) AUC values following binary logistic regression analysis of indicated serum markers are shown. (Red to green color indicates highest to lowest prognostic power or AUC value)

**Fig. 4:**
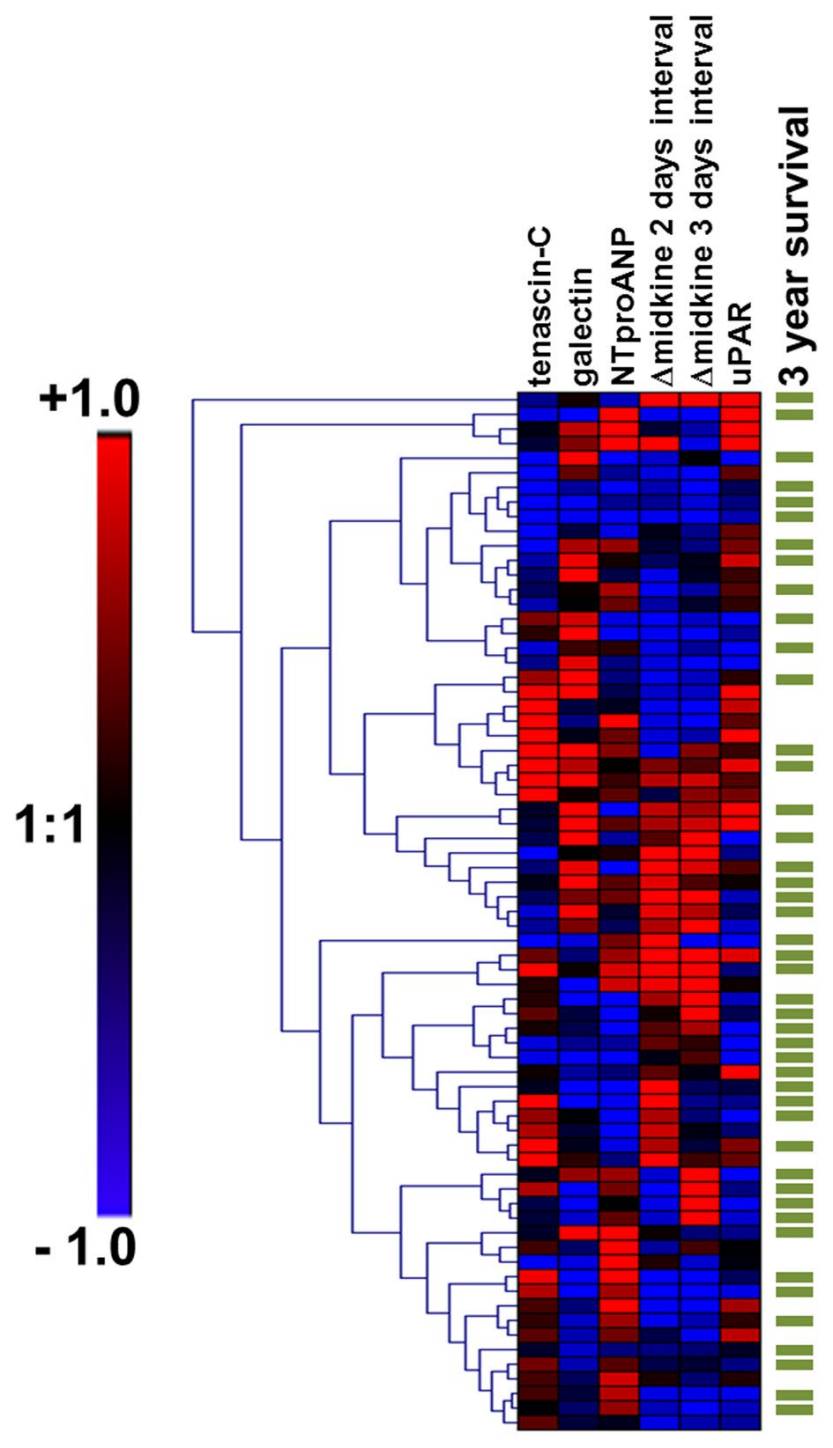
Heatmap analyses of serum marker determinations to identify subgroups of patients with highest risk scores and adverse outcome Genesis software was used for heatmap analysis and clustering of patients according to differentially expressed serum markers. *X* axis represents serum markers and *Y* axis patients. Visualization was done using Z-score transformation of the raw data followed by hierarchical clustering of patients with average linkage as agglomeration rule. Red and blue colours indicate higher or lower than average expression levels, as visualized by the scale bar. Patients surviving 3 years are indicated by green line to the right.

Clustering of the biomarker and midkine results was performed using Genesis software (Fig. 4). By selecting patients that deceased within a 36 month observation period we were able to visualize possible patient subcohorts with poor cardiovascular outcome. The heatmap demonstrates the high replicability of the Δmidkine changes in individual patients, links a “lower than average” increase of midkine (blue color) with elevated uPAR serum levels, and poor outcome. In contrast tenascin-C, galectin, and NTproANP cluster separately (Fig. 4).

For 81 patients, the plasma levels of ADMA, SDMA, and L-arginine in pre-dialysis samples were determined (Suppl. Table 2). For those patients Kaplan–Meier survival curves were plotted with ADMA values above/below average, exhibiting no significant differences in (cardiovascular) mortality over 36 months (Suppl. Fig 6A, D). Furthermore, average ADMA, SDMA, and L-arginine serum values were determined for patients with Δmidkine values above/below average, again without significant differences (not shown), suggesting that midkine release and basal ADMA, SDMA, and L-arginine metabolism are not interconnected.

**Fig. 5:**
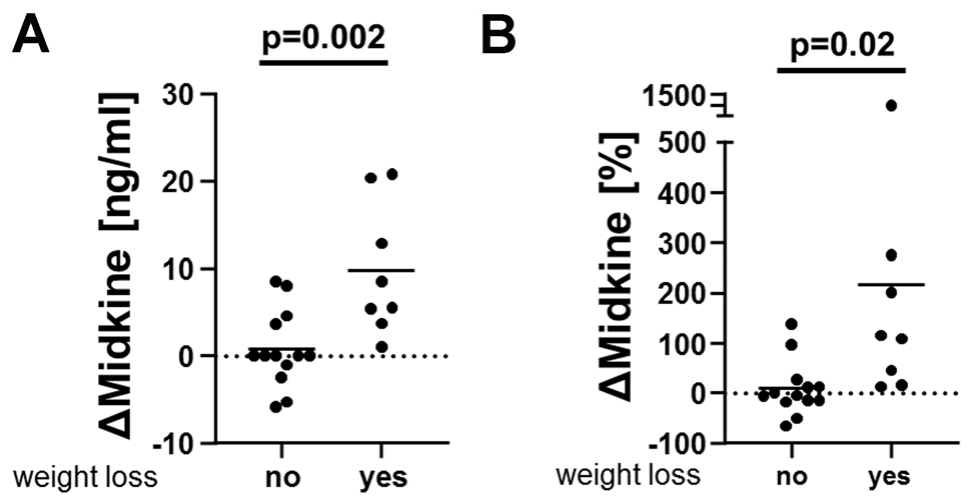
Effect of fluid removal on midkine release in patients with hypervolemia. In patients diagnosed with hypervolemia (*n* = 21) appropriate fluid management was planned by increasing net fluid removal during dialysis sessions. Serum midkine levels after long dialysis-free intervals were quantified. 8/21 patients successfully removed additional 0.5–1.0 kg (*n* = 3) or > 1.0 kg (*n* = 5). **A** ∆Midkine levels were calculated as maximal difference to baseline in the 3 week intervention period. **B** Change of midkine values calculated as [%] change to baseline values

Finally, an intervention study with 21 hypervolemic dialysis patients (V. cava width, comet tail, edema formation, fluid retention within the lungs) was set up. Additional fluid removal by dialysis procedures was recommended and, whenever tolerated, removed by dialysis in a stepwise manner over 3 weeks. Blood sampling pre- and post-procedural was performed following long intervals was performed for midkine determinations. 8/21 patients successfully reduced their excessive fluid, at the same time circulating midkine levels increased by 8.9 ± 2.5 ng/ml (+ 208.1 ± 87.8% versus patients w/o fluid removal). The data indicate that midkine release is linked with fluid homeostasis and correction of body fluid composition, constituting a natural regulatory response (Fig. 5, Suppl. Fig. 7).

## Discussion

End-stage renal disease and dependency on chronic renal replacement therapy goes along with an extremely high cardiovascular risk profile, which reaches a yearly mortality rate up to > 20% [23]. The Dialysis Outcomes and Practice Patterns Study (DOPPS) indicates a higher mortality rate in diabetic patients undergoing hemodialysis, which reached 16% in a recent survey [24], mostly due to cardiovascular events. These often originate in episodes of cardiac arrhythmias resulting in sudden cardiac death [25].

One of the striking findings is that after a longer dialysis-free interval, which takes mostly 3 days after the weekend, excessively higher mortality rates are observed [26, 27]. These observations led to the hypothesis that beyond elevated uremia toxins clinically relevant fluid and electrolyte imbalances may contribute to adverse outcomes [25]. Fluid imbalances per se are a tedious and serious challenge to patients and caring physicians, often resulting into conflicts about compliance to advised fluid and sodium intake restrictions [28]. Imbalances of fluid homeostasis may also be the result of hormonal dysregulations, which orchestrate fluid distribution within the body, especially lungs, and are relevant to the fluid mobilization during a dialysis session. One of the hormonal candidates involved in fluid homeostasis is the heparin-binding growth factor midkine, which is synthesized and released by endothelial cells and modifies the synthesis and activities of angiotensin II within the lung vasculature [5].

Our primary hypothesis was that midkine release in dialysis patients is altered due to the dialysis procedure per se with preexisting generalized endothelial cell damage due to an inflammatory milieu [29]. Our data clearly link the strong increase in midkine with application of non-fractionated heparin as the anticoagulant, however heparin alone may not be responsible for this response. We observe a differential response to heparin. The dose of non-fractionated heparin correlates with the rise in midkine (*r*^2^ = 0.33), whereas fractionated heparin does not. This may be due to structural differences between the two [30]. It is likely that non-fractionated heparin molecules, which have more variable binding sites, enhance midkine expression, release or prolong its half-life. The observed differential effects of fractionated and non-fractionated heparin may anticipate distinctive long-term outcomes and should prompt additional investigations.

A positive fluid balance resulting in signs of hypervolemia is a common finding in chronic kidney disease (CKD). Fluid overload is associated with arterial hypertension, left ventricular hypertrophy, and accelerated arteriosclerosis. The dialysis procedure itself with fluid removal constitutes a cardiovascular stress that exceeds standard diagnostic adenosine stress testing or exercise electrocardiography [31]. Therefore maintaining an euvolemic state is important for preventing cardiovascular complications [32]. Fluid has to be mobilized by the body within a short period of dialysis time and prolonged fluid redistribution takes place especially within the interdialytic intervals. In our study, 63% of the patients were classified as hypervolemic and we found a strong negative correlation between hypervolemia and midkine values post dialysis. When the mean midkine level post dialysis was lower than average a fluid overload was more likely present, suggesting a link between fluid mobilisation and midkine release. In patients with clinical signs of hypervolemia midkine levels rose by about 6–8.9-fold, whereas in the absence of hypervolemia this rise was > 12.2–14.6-fold. Thus, vascular stress caused by chronic hypervolemia may suppress midkine release by endothelial cells or, conversely, a diminished midkine release may be causatively linked with a failure to mobilize interstitial fluid. One may envision clinical studies that address this point by adjusting the fluid removal according to Δmidkine levels that should rise at least 12.2-fold. Such an intervention may discern cause and effect of fluid disbalances and periprocedural changes of midkine levels. Further, the midkine response of healthy individuals subjected to fluid load/depletion would be of interest.

One may speculate that endogenous clearance of midkine through diuresis might have an effect of circulating levels, however our pre-dialysis determinatins revealed only minor differences in healthy controls versus dialysis patients. Thus, a major influence by diuresis can be excluded and the extent of fluid removal and the individual fluid homeostasis are likely affecting the variability of midkine changes. Our data sets furthermore indicate that the effect of heparin, as fractionated or non-fractionated fabrication, do not have an effect on circulating midkine levels (Suppl. Fig 2).

Serum midkine levels in healthy subjects are mostly determined within a narrow range that depends on the applied detection system. Beyond these “background” levels, several diseases are known to be accompanied by elevated midkine serum levels, e.g., cancers of different origin, ischemia of brain, limbs or kidneys [33], and congestive heart failure [4, 34–37].

The main challenge to the use of midkine as diagnostic biomarker by single determinations is the generality of its regulation and lack of specificity for particular diseases [34]. To address this issue and not rely on single serum levels determined in index patients our study protocol comprised of serial midkine determinations, performed before and after dialysis sessions, repeated on two occasions and with differing fluid removal.

The sources of midkine and mechanisms of release are not well understood [38]. The lung endothelium may constitute a major source, given that a vicious cycle of positive feedback in the midkine—angiotensin II pathway with oxidative stress and subsequently up-regulated ACE exists [19]. Kidney-lung interactions might partly account for the pathogenesis of hypertension and kidney damage; however these have not been analyzed in detail in dialysis patients with prevailing kidney fibrosis. In a small study, a stimulatory effect of heparin application on serum midkine release in dialysis patients and healthy controls was determined [12], whereas other studies quantified midkine and inflammatory cytokine release in sepsis and vascular disease [39, 40].

A striking finding of our study is the predictive association of midkine release for (cardiovascular) mortality in dialysis patients. When the rise of serum midkine values during dialysis was below average, the likelihood of cardiovascular mortality was significantly increased; a finding that becomes even more apparent when the upper 75 and lower 25 percentiles are compared. Thus, following a long dialysis-free interval testing for midkine levels before and after dialysis may be suitable for risk stratification and adjustment of optimal weight. Clearly, this testing outperforms other markers for endothelial dysfunction, such as galectin [21] and tenascin-C [22], as well as ADMA, which is controversially discussed as mortality predictor [41, 42].

Furthermore one might question how serum midkine levels may be therapeutically modified and whether midkine is the missing link to explain fluid retention and dysregulation in hypertensive patients. The latter is of paramount importance for dialysis patients, where non-adherence to “fluid recommendations” is daily practice and a less-than-appropriate midkine release may possibly explain fluid retention and hyperhydration. The performed intervention study with additional fluid removal supports that notion that midkine release is directly linked with fluid removal and body homeostasis. One of the clinically relevant aspects is that blood pressure changes are not always correlated with fluid changes [43]. One may envision that with adequate midkine release arterial hypertension develops, given that midkine also propagates angiotensin synthesis [38]. In some patients blood pressure remains low, even with fluid excess and may decrease at the end of dialysis sessions, when the body fluid removal peaks. It will be of interest whether midkine is similarly regulated in patients with cardiac failure and/or vascular diseases undergoing a stress test, even in the absence of kidney malfunction.

Recombinant human midkine is available and enters clinical studies [44], e.g., to combat heart failure. Possibly, another field of applicability would be the subgroup of dialysis patients that suffer from excess fluid retention, do not respond to diuretics due to failing excretory kidney function and that have a suboptimal release of midkine for fluid mobilization.

The main limitation of the study is the size of the primary study cohort. We have therefore enrolled a second independent cohort and confirmed the results. The midkine release test may have a far reaching predictive character, may shed a novel light on fluid retention in dialysis patients. Furthermore, this study follows an observational design and cannot demonstrate a direct cause-and-effect risk associations.

## Supplementary Information

Below is the link to the electronic supplementary material.

Supplementary file1 (PDF 1624 KB)
