# Supplementary material for: Midkine release during hemodialysis is predictive of hypervolemia and associates with excess (cardiovascular) mortality in patients with end-stage renal disease: a prospective study
Source: Int Urol Nephrol. 2022 Feb 24;54(9):2407–20. doi: 10.1007/s11255-022-03141-4 (PMC9372127; doi:10.1007/s11255-022-03141-4)
Supplement: Supplementary file 1 — Supplementary file1 (PDF 1624 KB) [file 11255_2022_3141_MOESM1_ESM.pdf]

Supplementary material

Supplementary Figure 1: Midkine stability is ascertained by storage at -80°C and preclusion of repeated freeze-thaw cycles

Supplementary Figure 2: Correlation of applied heparin with delta midkine levels

Supplementary Figure 3: Serum midkine levels before and after dialysis treatment

Supplementary Figure 4: Kaplan-Meier survival curves for tenascin-C and galectin

Supplementary Figure 5: Correlative analyses of midkine, NTproANP and uPAR

Supplementary Figure 6: Kaplan-Meier survival curves for ADMA

Supplementary Figure 7: Midkine release in patients with hypervolemia.

Supplementary Table 1: Fluid homeostasis assessment

Supplementary Table 2: ADMA, SDMA, DGMV, L-arginine serum levels did not correlate with delta midkine serum levels

Supplementary Table 3: Hazard Ratio for ΔMidkine, uPAR, and ANP adjusted for age, gender, fluid status, and co-morbidity diabetes.

Supplementary Figure 1: Midkine stability is ascertained by storage at -80°C and preclusion of repeated freeze-thaw cycles. (A) Serum samples from 3 different patients were stored at 4°C, -20°C, or -80°C. Quantification of midkine was carried out after 4 days following immediate thawing before performance of the ELISA. **(B)** Serum samples from the same patients were stored at 4°C, -20°C, or -80°C for four days. Before quantification by midkine ELISA samples were thawed and subjected to another freezing/thawing cycle (1st thawing) and two more freezing/thawing cycles (2nd thawing). **(C)** Long term stability was tested with serum samples from two patients that were included for ELISA quantification after 2 and 3 years storage at -80°C.

Supplementary Figure 2: Correlation of applied heparin with delta midkine levels. (A) The doses of applied non-fractionated heparin were correlated with changes in serum midkine levels after a short ($r^2=0.06$, $p=0.03$) and long dialysis-free interval ($r^2=0.17$, $p<0.001$). **(B)** For non-fractionated heparin similar correlations of delta midkine levels after a short ($r^2=0.002$, $p=0.88$) and long dialysis-free interval ($r^2=0.01$, $p=0.76$) were performed in 12 patients (average 2,958 ± 1,389 IU). The increase of midkine levels was similar after both intervals, 17.6 ± 17 ng/ml and 14.5 ± 11.9 ng/ml, respectively, and did not positively correlate with the dose of fractionated heparin.

Supplementary Figure 3: Serum midkine levels before and after dialysis treatment. (A) Δmidkine values (midkine post – pre-hemodialysis) were calculated for the short and long interval, yielding no significant difference. Furthermore the ΔΔmidkine levels (Δmidkine short interval - Δmidkine long interval) were calculated to assess the variability of midkine changes. **(B)** For the subgroups of diabetic (n=33) versus non-diabetic (n=50) patients analyses were performed. Δmidkine (midkine levels after dialysis - before dialysis) were calculated for the short and long dialysis-free interval. **(C)** For the subgroups of patients diagnosed with hypervolemia (n=51) versus euvolemia (n=32), Δmidkine (serum midkine levels after dialysis - before dialysis) were calculated for the short and long dialysis-free interval. Here, significant differences for the subgroup comparisons were confirmed. **(D)** ROC curve analyses showing the prognostic power of absolute midkine values or Δmidkine values to predict diabetes (cohort with n=70 diagnosed with diabetes; n=101 without diabetes),

hypervolemia (cohort with n=83 diagnosed with hypervolemia; n=88 without hypervolemia). In the last panels prediction of both conditions combined is tested (cohort with n=32 diagnosed with diabetes and hypervolemia; n=51 without diabetes andhypervolemia). AUC, area under the curve. **(E)** ROC curve analyses showing the prognostic power of Δmidkine values with binary logistic regression performed following short and long dialysis-free intervals to diagnose hypervolemia. Selected cut-off points for Δmidkine of the short and long intervals are indicated.

Supplementary Figure 4: Kaplan-Meier survival curves for tenascin-C and galectin. Serum concentrations of tenascin-C **(A, B)** and galectin **(C,D)** were determined. Kaplan-Meier survival curves indicate that both parameters are not predictive for overall survival.

Supplementary Figure 5: Correlative analyses of midkine, NTproANP and uPAR. NTproANP and uPAR serum concentrations were correlated (Pearson) with absolute midkine serum values in the whole dialysis cohort **(A,C)** or selected patients diagnosed with diabetes **(B,D)**.

Supplementary Figure 6: Kaplan-Meier survival curves for ADMA. (A) Patients with less-than-average ADMA serum levels before the long dialysis-free interval (group 1) were compared with those having above-average ADMA levels (group 2). Over a 36 months observation period, censoring was performed for overall mortality and **(B)** cardiovascular mortality.

Supplementary Figure 7. Effect of fluid removal on midkine release in patients with hypervolemia. In patients diagnosed with hypervolemia (n=21) appropriate fluid management was planned by increasing net fluid removal during dialysis sessions. Serum midkine levels after long dialysis-free intervals were quantified. 8/21 patients successfully removed additional 0.5-1.0 kg (n=3) or >1.0 kg (n=5). **(A)** ΔMidkine levels were calculated as maximal difference to baseline in the 3 week intervention period with subgrouping according to weight loss: >0.5 to 1.0 kg and >1.0 kg. **(B)** Change of midkine values calculated as [%] change to baseline values again with subgrouping according to weight loss: >0.5 to 1.0 kg and >1.0 kg.

Supplementary Figure 1

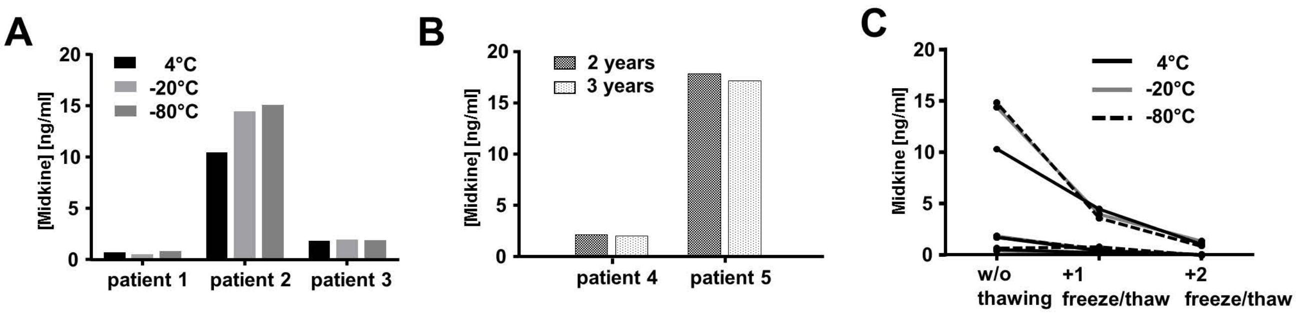

Supplementary Figure 2

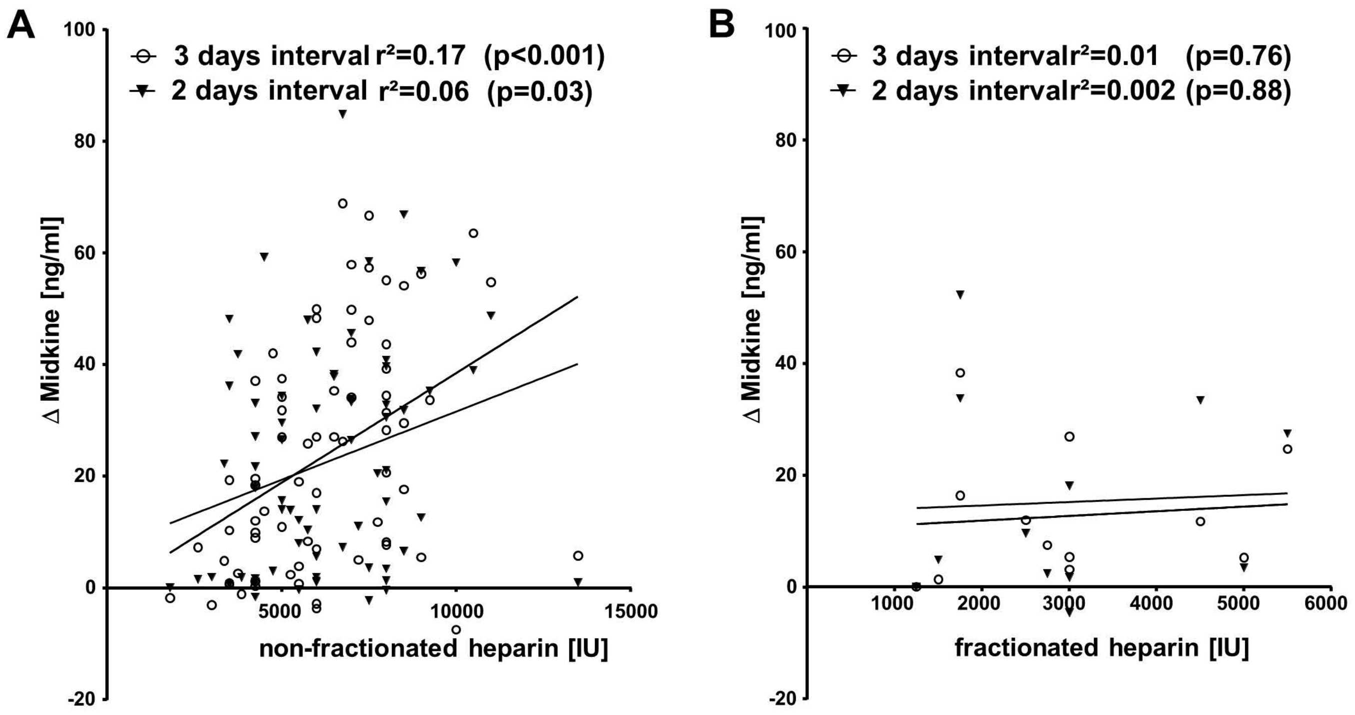

Supplementary Figure 4

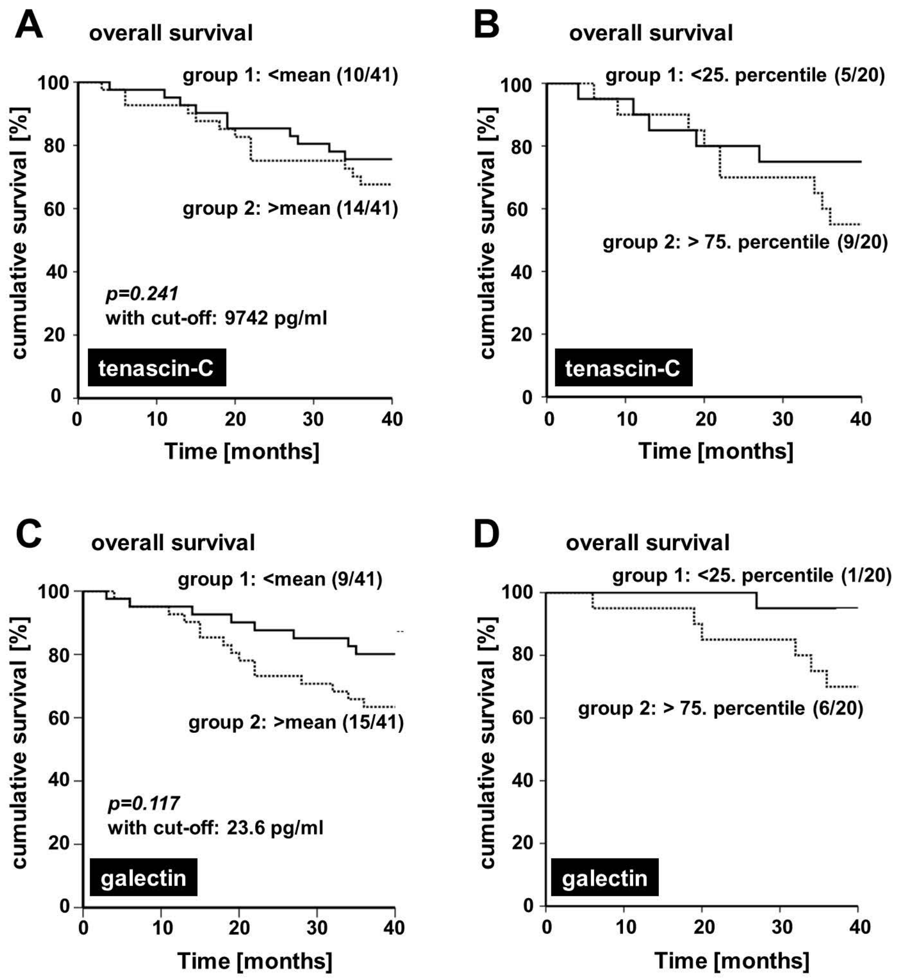

Supplementary Figure 3

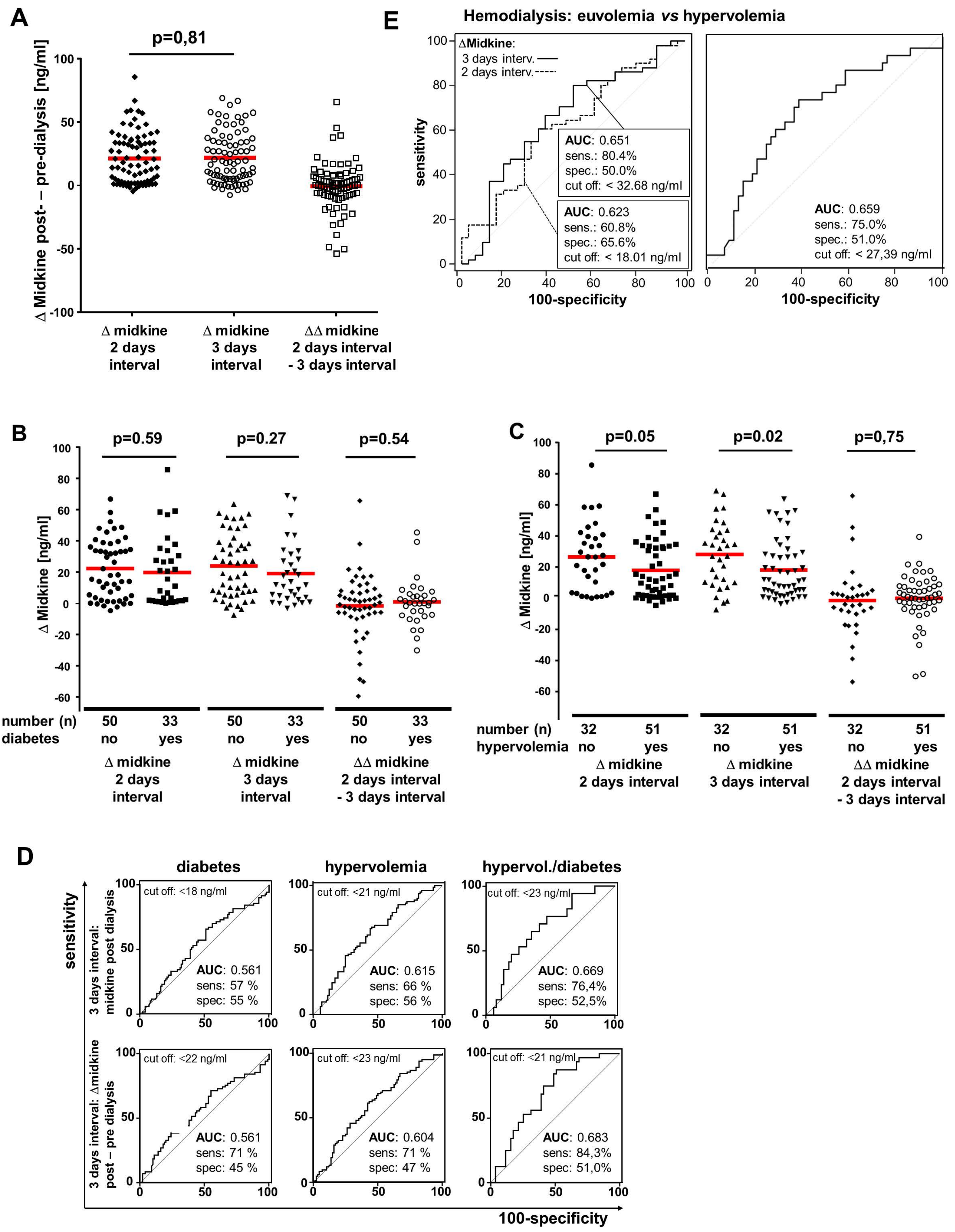

Supplementary Figure 5

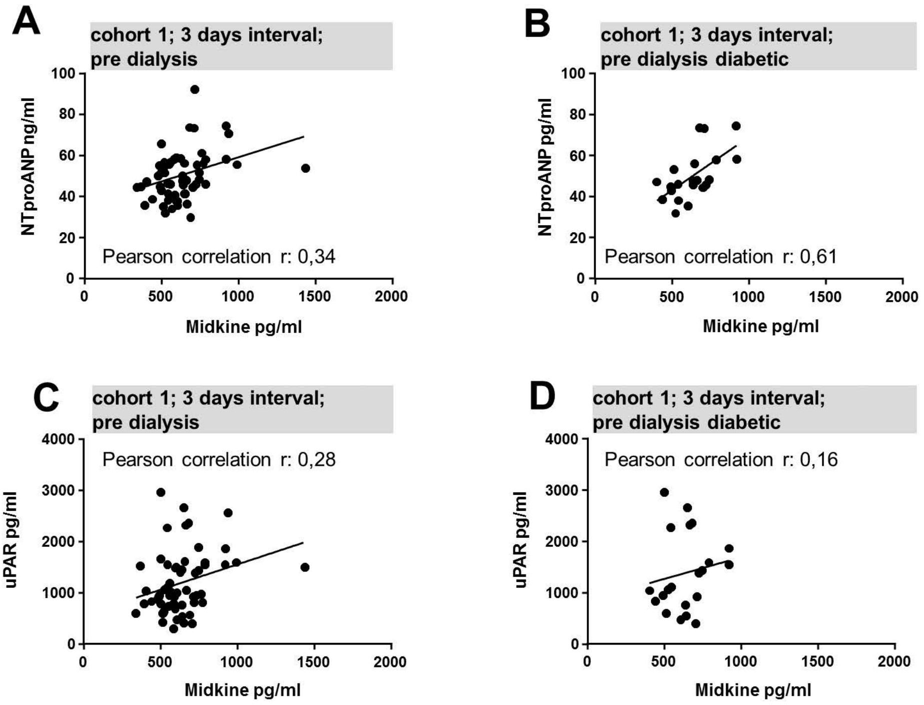

Supplementary Figure 6

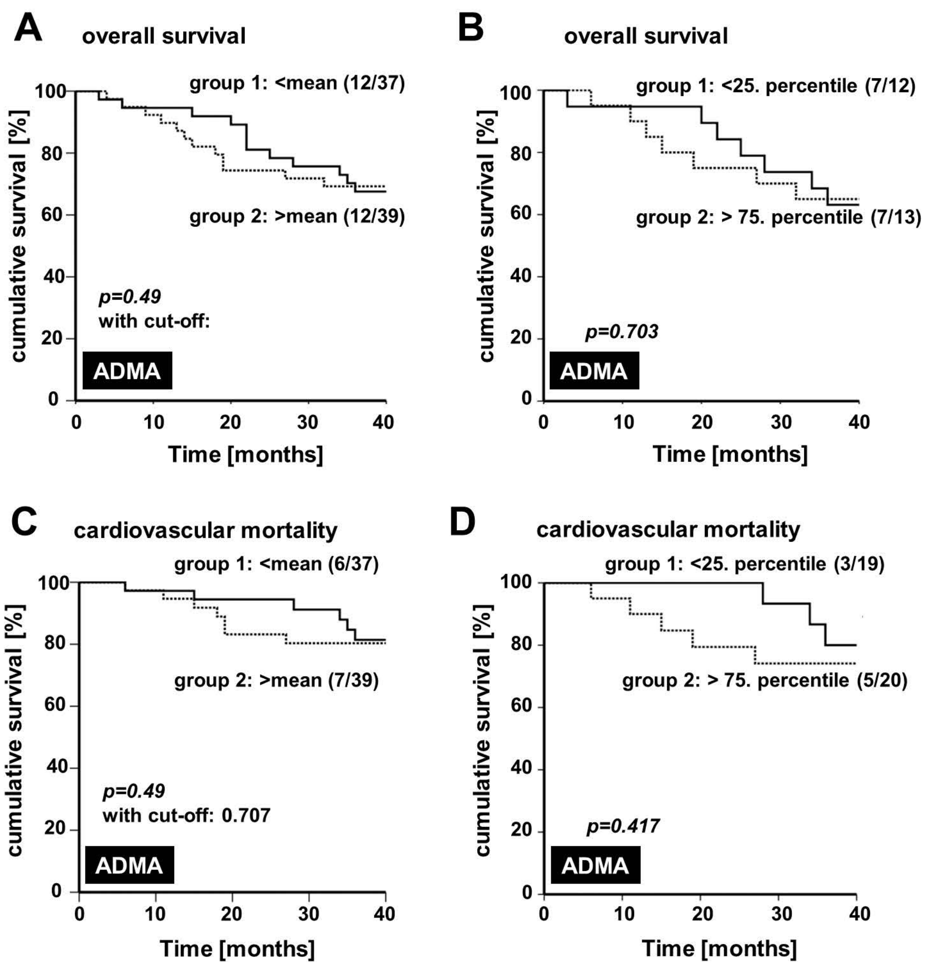

Supplementary Figure 7

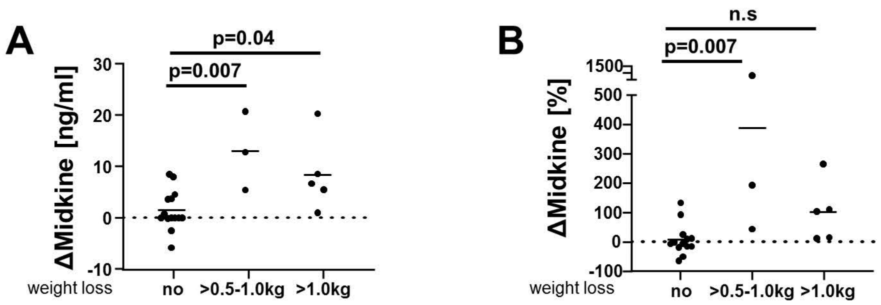

Supplementary Table 1

Fluid homeostasis assessment

	hypervolemia defined by		
	comet tail sign lungs	elevated weight (> +0.5 kg)	V. cava diameter > 20mm
**number of patients (n)**	11	38	22

Supplementary Table 2

ADMA, SDMA, DGMV, L-arginine, delta midkine serum levels

Delta Midkine	3 days interval above mean [24,9 ng/ml] n=42	3 days interval below mean [24,9 ng/ml] n=41	p-value
ADMA [μmol/l]	0.49 ± 0.99	0.49 ± 0.09	0.88
SDMA [μmol/l]	1.28 ± 0.36	1.33 ± 0.1	0.65
DGMV [mmol/l]	334.96 ± 218.34	356.07 ± 385.85	0.77
L-arginine [μmol/l]	22.78 ± 9.4	24.11 ± 20.7	0.72

Supplementary Table 3. HR for ΔMidkine, uPAR, and ANP adjusted for age, gender, fluid status, and diabetes. (*Abbreviations: CI, confidence intervall; HR, hazard ration.)*

	**HR**	**95% CI**
**ΔMidkine**		
HR_{Age}	**1.059**	**1.026 - 1.094**
HR_{Gender}	0.905	0.447 - 1.830
$HR_{Hypervolemia}$	1.367	0.652 - 2.863
$HR_{Diabetes}$	0.898	0.457 - 1.762
$HR_{\Delta Midkine}$	**2.204**	**1.244 - 3.641**
**uPAR**		
HR_{Age}	**1.052**	**1.017 - 1.088**
HR_{Gender}	1.083	0.533 - 2.199
$HR_{Hypervolemia}$	1.720	0.824 - 3.592
$HR_{Diabetes}$	0.987	0.501 - 1.943
HR_{uPAR}	**2.421**	**1.153 - 5.086**
**ANP**		
HR_{Age}	**1.055**	**1.022 - 1.090**
HR_{Gender}	0.951	0.468 - 1.934
$HR_{Hypervolemia}$	1.729	0.833 - 3.590
$HR_{Diabetes}$	1.036	0.529 - 2.029
HR_{ANP}	1.352	0.678 - 2.697
